# A comparative study on the analysis of hemodynamics in the athlete’s heart

**DOI:** 10.1038/s41598-022-20839-8

**Published:** 2022-10-05

**Authors:** Utku Gülan, Valentina A. Rossi, Alexander Gotschy, Ardan M. Saguner, Robert Manka, Corinna B. Brunckhorst, Firat Duru, Christian M. Schmied, David Niederseer

**Affiliations:** 1 Hi-D Imaging, 8406 Winterthur, Switzerland; 2 Department of Cardiology, University Heart Center, University Hospital Zurich, Rämistrasse 100, 8091 Zurich, Switzerland; 3 Center for Integrative Human Physiology, University of Zurich, Zurich, Switzerland

**Keywords:** Cardiology, Blood flow

## Abstract

The pathophysiological mechanisms underlying the development of the athlete’s heart are still poorly understood. To characterize the intracavitary blood flows in the right ventricle (RV) and right-ventricular outflow tract (RVOT) in 2 healthy probands, patients with arrhythmogenic right ventricular cardiomyopathy (ARVC) and 2 endurance athletes, we performed 4D-MRI flow measurements to assess differences in kinetic energy and shear stresses. Time evolution of velocity magnitude, mean kinetic energy (MKE), turbulent kinetic energy (TKE) and viscous shear stress (VSS) were measured both along the whole RV and in the RVOT. RVOT regions had higher kinetic energy values and higher shear stresses levels compared to the global averaging over RV among all subjects. Endurance athletes had relatively lower kinetic energy and shear stresses in the RVOT regions compared to both healthy probands and ARVC patients. The athlete’s heart is characterized by lower kinetic energy and shear stresses in the RVOT, which might be explained by a higher diastolic compliance of the RV.

## Introduction

Extensive training results in repetitive volume and pressure overloads, which are responsible for cardiac remodeling involving both ventricles. The right ventricle (RV) is particularly susceptible to exercise-related hemodynamic variations, thus leading to a structural, functional and electrical remodeling^1,2^. The morphologic remodeling in the so-called *athlete’s heart* and the RV alterations described in arrhythmogenic ventricular cardiomyopathy (ARVC) significantly overlap, although the underlying mechanisms significantly differ^3^. ARVC is a polygenic disease with different penetrance and degree of gravity involving desmosomal mutations^4^. There is evidence suggesting that intensive exercise may not only accelerate the development of an adverse RV remodeling in affected patients, but may also induce cardiac fibrosis and a pathologic RV dilation, leading to an increased risk for malignant arrhythmia even in healthy subjects^5^.

The analysis of fluid dynamics parameters such as mean kinetic energy (MKE), turbulent kinetic energy (TKE) and viscous shear stress (VSS) as measured by MRI have already been tested as hemodynamic indicators of severity in Tetralogy of Fallot, aortic valve stenosis and mitral regurgitation^6–8^.

The aim of this study is to better understand the hemodynamic differences of the blood flow in RV underlying the different RV remodeling pattern in endurance athletes and ARVC patients compared to healthy subjects.

## Methods

### Study population

In this observational study, male subjects were categorized into 3 groups as follows: healthy volunteers with mild daily activities (Healthy group, n = 2), athletic male probands with endurance sport activity history (Athlete group, n = 2), and patients with definite ARVC according to the 2010 Task Force Criteria (ARVC group, n = 2)^9,10^. Athletes underwent MRI examination during their regular training and cardiopulmonary exercise testing showed a VO_2peak_ of 37 ml/min/kg, corresponding to 339 W, 175% of theoretical maximal capacity, and a VO_2peak_ of 43 ml/min/kg, corresponding to 412 W, 197% of theoretical maximal capacity, respectively. ARVC patient #1 was diagnosed during regular health check-up and presented with major imaging criteria and repolarization abnormalities. ARVC patient #2 presented initially with palpitation during sport activity and was diagnosed with major arrhythmia criteria due to the finding of ventricular tachycardia. Both ARVC patients did not perform high-intensity sport neither at the moment of ARVC diagnosis, nor before, but only moderate-intensity leisure activity^11^. Their functional capacity at exercise testing revealed a good functional capacity of 206 W, corresponding to 99% of the theoretical maximal capacity, and of 239 W, corresponding to 111% of the theoretical maximal capacity, respectively.

All patient groups were screened and recruited for 4D flow MRI scans at the University Hospital of Zurich. Informed consent was obtained for all participants. The study conformed to the principles outlined in the Declaration of Helsinki and was approved by the local Ethical Committee of Canton Zurich (EK-Nr. PB_2016-02109).

### In vivo MRI measurements

All subjects were scanned with a 3T Philips Ingenia System (Philips Healthcare, Best, The Netherlands). For 4D Flow imaging, a spoiled gradient echo sequence with multipoint velocity encoding using 10 different velocity encodings (3 per spatial direction plus 1 reference encoding) was applied. The imaging parameters were as follows: spatial resolution 2.5 × 2.5 × 2.5 mm^3^, field of view 250 × 160 × 50 mm^3^. The velocity encoding (VENC) values were 40, 100 and 200 cm/s per direction. More technical details can be found in the previous publications of Gülan et al.^12,13^. The heart rate and cardiac output at the moment of the MRI analysis are reported in Table 2. All the subjects who underwent the investigation were in a clinically euvolemic status, with no signs nor symptoms of a concomitant infection.

### Flow field analysis

The anatomical segmentation was performed using an in-house algorithm in MATLAB. Four different hemodynamics parameters, namely velocity, mean kinetic energy (MKE), turbulent kinetic energy (TKE) and viscous shear stress (VSS) were utilized as an indicator of the hemodynamics performance in the circulatory system. The voxelwise total kinetic energy can be decomposed into mean kinetic energy (MKE) and turbulent kinetic energy (TKE). The mean kinetic energy (MKE) is calculated for each voxel for different phases of the cardiac cycle^12,13^ as half of the blood density times the square of the phase averaged velocity:

1 $$MKE=\frac{\uprho }{2}\overline{{U }_{i}}\overline{{U }_{i}},$$

where ρ is the blood viscosity, $\overline{{U }_{i}}$ is the three dimensional phase averaged velocity, and I is the velocity component. Similarly, TKE can be calculated for each voxel for different phases of the cardiac cycle as the sum over the three spatial axis of the product of half the blood density and the square of the fluctuating velocity:

2 $$TKE=\frac{\uprho }{2}\sum_{i=1}^{3}{{{u}_{i}}^{{\prime2}}},$$

where ${{u}_{i}}^{^{\prime}}$ is the three dimensional fluctuating velocity and i is the velocity component.

Finally, viscous shear stresses (VSS) are calculated as the gradient of the mean velocity profile:

3 $$VSS=\mu \left(\frac{\partial \overline{{U }_{i}}}{\partial {x}_{j}}+\frac{\partial \overline{{U }_{j}}}{\partial {x}_{i}}\right),$$

where μ is the dynamic viscosity of the fluid, as previously described^13^.

### Echocardiographic measurements

RV and LV end-diastolic diameters indexed for body surface area were measured in parasternal long axis according to current guidelines^14^.

## Results

The baseline characteristics of the male subjects are summarized in Table 1. Intracavitary blood flow parameters obtained via 4D-MRI in vivo for all patient are summarized in Table 2.

**Table 1:** Baseline characteristics.

Parameters	Healthy#1	Healthy#2	Athlete#1	Athlete#2	ARVC#1	ARVC#2
Age (years)	52	35	54	37	53	30
Body surface, m^2^	1.77	2.01	2.16	1.94	1.88	1.68
Co-morbidities	–	–	–	–	–	Isolated mild aortic dilation
Medications	–	–	–	–	–	Metoprolol 25 mg/day
Type of sport activity	–	–	Cycling	Ironman triathlon	Jogging and cycling moderate intensity	Soccer
Training hours/week	–	–	14 h/week	16 h/week	3 h/week	3 h/week
Exercise test	–	–	Spiroergometry 339 W, 175%max, RER 1.11	Spiroergometry 412 W, 197%max, RER 1.44	Ergometry 206 W, 99%max, DPF 4.4	Ergometry 239 W, 111%max, DPF 5.4

*DPF* double-product factor, *RE* respiratory exchange rate.

**Table 2:** Flow parameters obtained in vivo for all study participants.

Parameters	Unit	Healthy#1	Healthy#2	Athlete#1	Athlete#2	ARVC#1	ARVC#2
Body surface	m^2^	1.77	2.01	2.16	1.94	1.88	1.68
RV ejection fraction	%	53	47	49	52	42	42
RV EDV	ml	170	204	278	196	216	211
EDDi of RVOT	mm	17	16	18.5	16.5	17	15
Thickness of RVOT	mm	5	5	6	6	4.5	4
Heart beat	bpm	66	62	53	54	63	65
Stroke volume	ml	90.6	88.2	136	101	87.5	91.6
Cardiac output	Lt/min	5.98	5.31	7.21	5.45	5.51	5.95

*EDDi* end-diastolic diameter indexed for body surface area, *EDV* end-diastolic volume, *RV* right ventricular, *RVOT* right ventricular outflow tract.

### Velocity analysis

**Figure 1:**
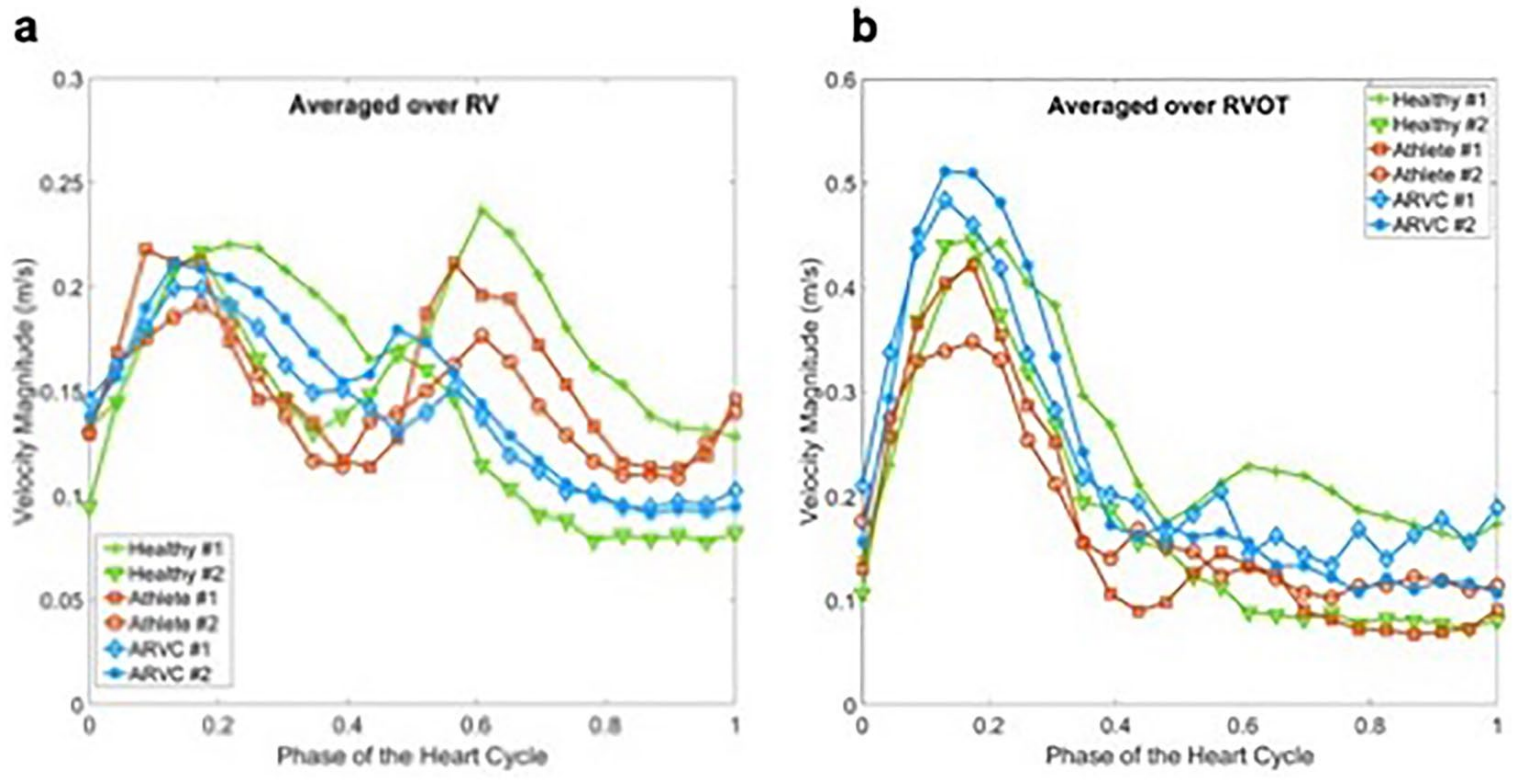
(**a**) Time evolution of velocity magnitude averaged over RV for healthy probands, athletes and patients with ARVC. (**b**) Time evolution of velocity magnitude averaged over RVOT for healthy probands, athletes and patients with ARVC.

Temporal evolution of velocity magnitude spatially averaged over RV is shown in Fig. 1a. The temporal trends of blood velocity in healthy probands, athletes and patients with ARVC are similar, i.e. an increase in systole due to high velocity regions in the vicinity of RVOT where RV forward the ventricular blood volume into pulmonary artery^7^ and a second peak in diastole due to the high velocity regions in the vicinity of the tricuspid region where the atrial blood flow fills the RV. Figure 1b depicts the velocity magnitude averaged over RVOT. The temporal trends is different from the ones averaged over the entire RV (Fig. 1a). During the ventricular contraction, i.e. systolic phase, higher velocities develop in the RVOT region. The ARVC patients show higher velocity regions during this phase of the cycle. In the diastolic phase, the magnitude of the velocity becomes smaller as compared to the systolic velocities.

### Kinetic energy analysis

**Figure 2:**
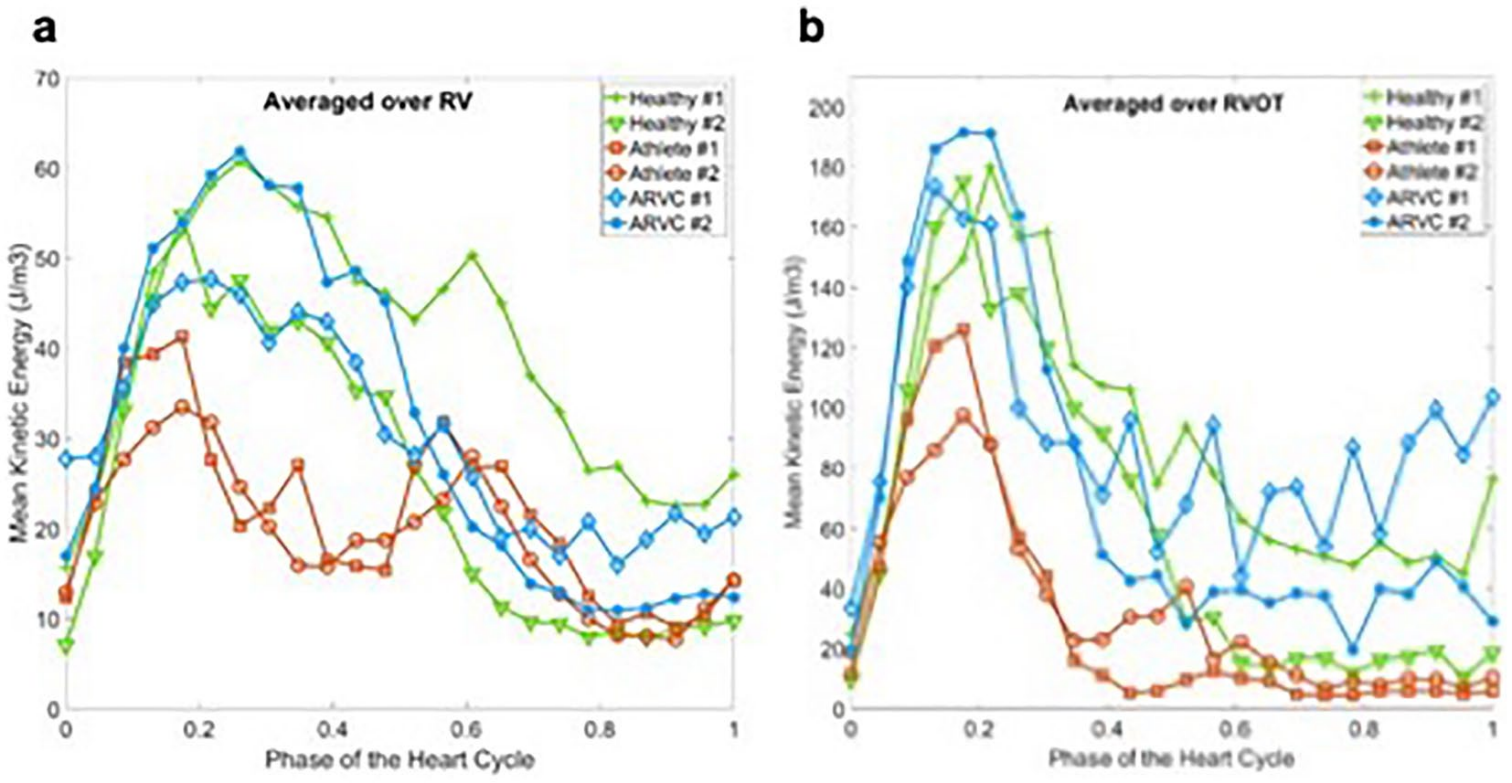
(**a**) Time evolution of mean kinetic energy (MKE) averaged over RV for healthy probands, athletes and patients with ARVC. (**b**) Time evolution of mean kinetic energy (MKE) averaged over RVOT for healthy probands, athletes and patients with ARVC.

Temporal evolution of MKE averaged over the entire RV is shown in Fig. 2a. The athletes show lower MKE during the systolic phase whereas the patients with ARVC develop higher MKE during the ventricular contraction phase. To have a better understanding on the spatial distribution of MKE, temporal evolution of MKE averaged over RVOT was depicted in Fig. 2b. As see, athletes show considerably lower MKE levels in the systolic phase compared to the patients with ARVC and healthy probands.

**Figure 3:**
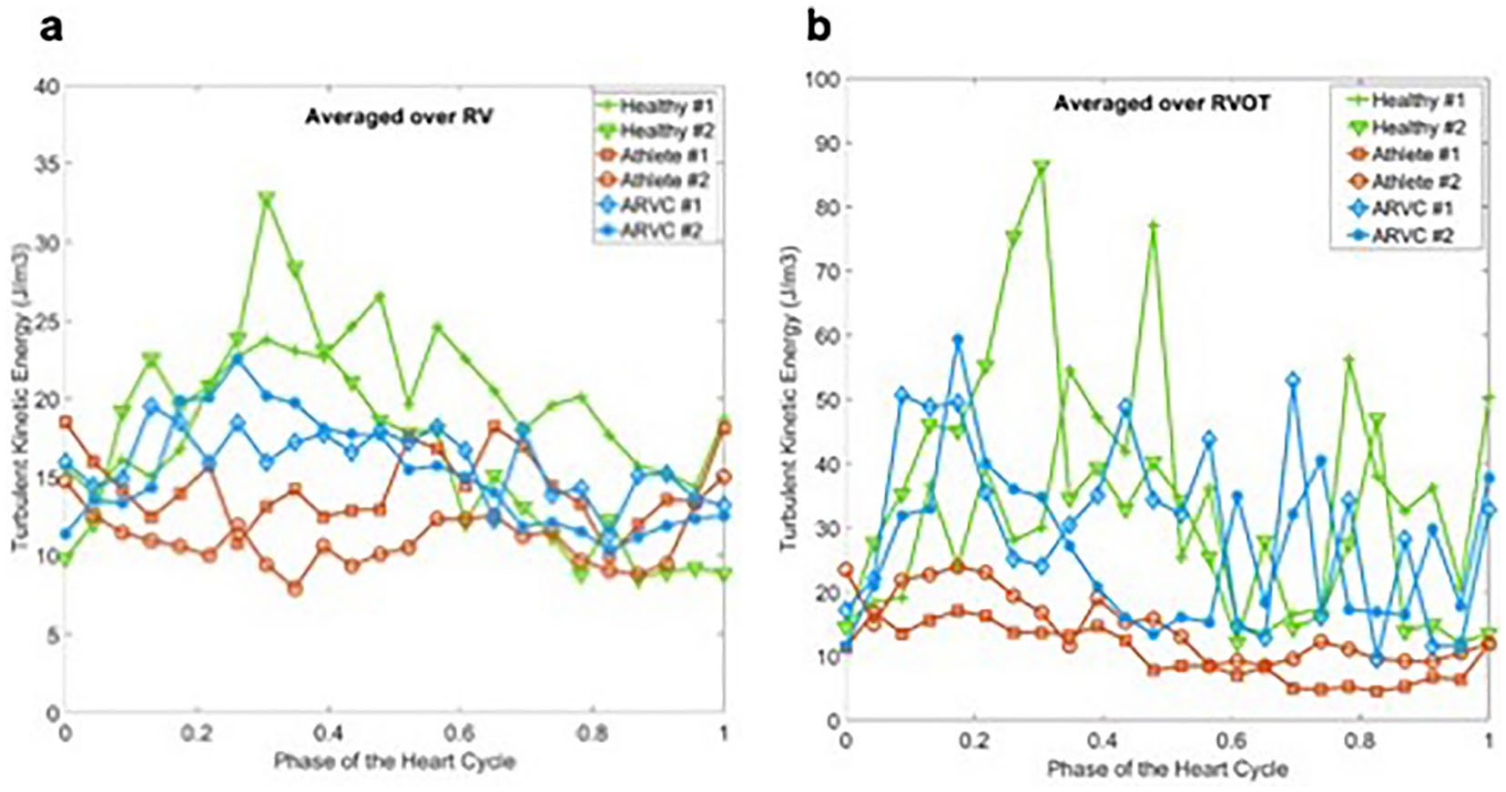
(**a**) Time evolution of turbulent kinetic energy (TKE) averaged over RV for healthy probands, athletes and patients with ARVC. (**b**) Time evolution of turbulent kinetic energy (TKE) averaged over RVOT for healthy probands, athletes and patients with ARVC.

Similar to the MKE analysis, temporal evolution of TKE averaged over the entire RV was presented in Fig. 3a. The temporal trends of TKE for all population vary during the heart cycle. The athletes show relatively lower TKE levels in late systole compared to the health probands and patients with ARVC. The TKE intensity variation during a heart cycle for all population is shown in Fig. 3b. Athletes show lower TKE values during the entire heart cycle. The healthy probands and patients with ARVC develop higher TKE regions compared to the athletes.

### Shear stress analysis

**Figure 4:**
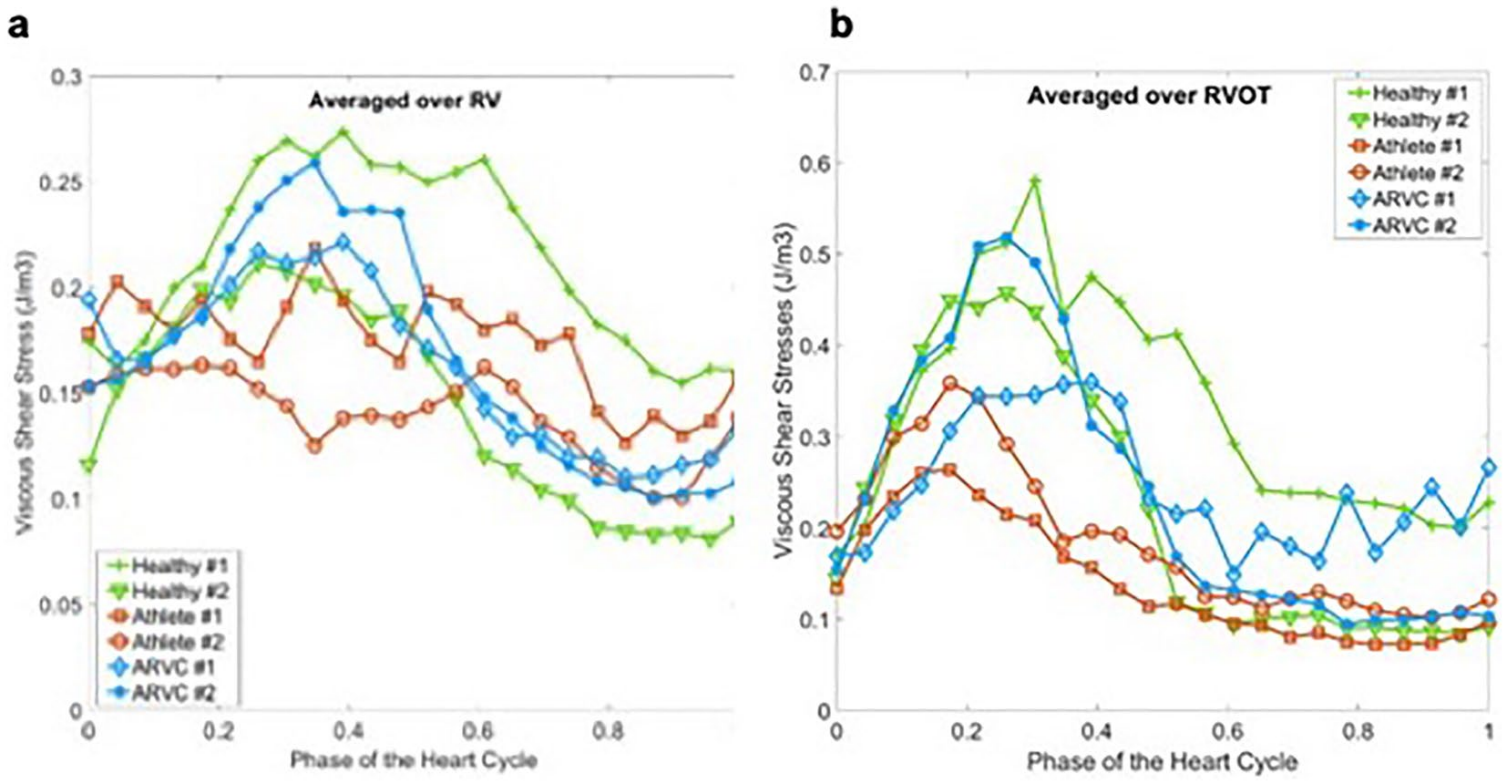
(**a**) Time evolution of viscous shear stress (VSS) averaged over RV for healthy probands, athletes and patients with ARVC. (**b**) Time evolution of viscous shear stress (VSS) averaged over RVOT for healthy probands, athletes and patients with ARVC.

Finally, the temporal trend of viscous shear stress is investigated. As shown in Fig. 4a, athletes show lower shear stresses in the systolic phase, yet higher stresses in the diastolic phase. Similar to Fig. 4a, temporal evolution of VSS averaged over the RVOT is depicted in Fig. 4b. Compared to the RV averaged stresses, RVOT averaged stresses show lower values for the athletes.

## Discussion

This study demonstrates time dependent, 3D blood flow patterns in the RV and RVOT as extracted using 4D-MRI flow measurements. To the best of our knowledge, this is the first study assessing the kinetic energy and shear stresses in the RV, and more specifically in the RVOT, by 4D-MRI in healthy probands, endurance athletes and ARVC patients.

In this study, we compared fluid dynamics of both RV and RVOT in healthy probands, endurance athletes and ARVC patients. RV and RVOT flow patterns have already been investigated in a previous study both in vivo and in vitro, showing that healthy probands and ARVC patients are subjected to higher MKE and WSS levels in the RVOT regions as compared to the rest of the RV^13^. The present data show that these findings apply to endurance athletes, as well: RVOT has higher kinetic energies and shear stresses compared to the global averaging over RV in all population analyzed. This finding might explain the high incidence of idiopathic tachycardias originating from the RVOT even in absence of structural heart disease^15,16^.

The diagnosis of ARVC is often challenging particularly at the early stages of disease, as it mostly involves young subjects. Furthermore, ARVC patients involved in both amateur and professional endurance sport activities are more likely to present with more severe clinical manifestations of ARVC. A better understanding of RV and RVOT fluid dynamics might help comprehend why genetically predisposed ARVC patients performing endurance activity develop a more severe phenotype and why otherwise healthy endurance athletes develop an ARVC-like phenotype even in absence of its corresponding genotype^5,17^.

Contrary to healthy probands and athletes, we found that ARVC patients experience in particular a second peak in flow velocity even during the diastole in the subtricuspid region, which is one of the first regions presenting with structural anomalies during the early stages of the disease.

Despite high RV and RVOT flow velocities, athletes had lower MKE values both along the whole RV and more specifically in the RVOT regions during both systole and diastole compared to ARVC patients. This finding likely reflects the differences in RV compliance, which is higher in athletes, whereas ARVC patients can develop RV diastolic dysfunction, which imply a negative prognosis^18^.

The analysis of blood stream turbulence was performed by assessing TKE, which has already been associated with adverse RV remodeling in patients with tetralogy of Fallot and relevant pulmonary valve regurgitation^8^. We found that athletes constantly showed lower TKE levels both along the whole RV and more specifically in the RVOT regions during all heart cycle phases compared to healthy probands and ARVC patients. This finding was confirmed also when shear stress originating from viscous fluid was analyzed. Our group previously demonstrated that there are higher shear stresses in the RVOT regions in a similar fashion in both healthy probands and ARVC patients^13^.

Interestingly we found that endurance athletes had both global RV and locally in the RVOT area lower shear stress values not only compared to ARVC patients, but also to healthy probands. The athlete’s heart is defined as a set of structural, electrical and functional remodeling as response to repetitive intense or extensive exercise. Endurance athletes present with four-chamber enlargement and higher biventricular mass in response to repetitive volume challenges^19–21^. Despite a resting biventricular systolic ejection fraction on the lower normal range, early diastolic function is usually normal to enhanced^22,23^. The current findings about lower RV shear stress in athletes might be related to a higher adaptation capability of the athlete’s heart to dynamic intracavitary stress related to blood flow, leading to an increased ventricular diastolic compliance.

### Limitations

The three groups investigated (healthy probands, athletes and ARVC patients) are represented only by two subjects each. Thus, interindividual variability could not be taken into account and formal statistical analyses were not possible. The proposed study reflects the observation of different physiological and pathological flow mechanics in these 3 groups and did not aim to provide definite answers, also because statistical analyses were not possible due to the low numbers. Our study can rather be regarded as hypothesis generating.

## Data Availability

The datasets generated and analyzed during the current study are not publicly available due to technical reasons but are available from the corresponding author on reasonable request.
